# Changes in Hepatobiliary Enzyme Abnormality After the Great East Japan Earthquake: The Fukushima Health Management Survey

**DOI:** 10.1038/s41598-017-00776-7

**Published:** 2017-04-06

**Authors:** Atsushi Takahashi, Tetsuya Ohira, Mayu Uemura, Mitsuaki Hosoya, Seiji Yasumura, Shigeatsu Hashimoto, Hiromasa Ohira, Akira Sakai, Akira Ohtsuru, Hiroaki Satoh, Yukihiko Kawasaki, Hitoshi Suzuki, Yoshihiro Sugiura, Hiroaki Shishido, Yoshimitsu Hayashi, Hideto Takahashi, Hironori Nakano, Gen Kobashi, Kotaro Ozasa, Hitoshi Ohto, Masafumi Abe

**Affiliations:** 1 Radiation Medical Science Center for the Fukushima Health Management Survey, Office of the Comprehensive Health Check and Health Promotion, Fukushima, Japan; 2 Fukushima Medical University School of Medicine, Fukushima, Japan; 3 Department of Gastroenterology, Fukushima Medical University, Fukushima, Japan; 4 Department of Epidemiology, Fukushima Medical University, Fukushima, Japan; 5 Department of Pediatrics, Fukushima Medical University, Fukushima, Japan; 6 Department of Public Health, Fukushima Medical University, Fukushima, Japan; 7 Department of Nephrology, Hypertension, Diabetology, and Endocrinology, Fukushima Medical University, Fukushima, Japan; 8 Department of Radiation Life Sciences, Fukushima Medical University, Fukushima, Japan; 9 Department of Radiation Health Management, Fukushima Medical University, Fukushima, Japan; 10 Department of Cardiology, Fukushima Medical University, Fukushima, Japan; 11 Department of Neurology, Fukushima Medical University, Fukushima, Japan; 12 Department of Orthopaedic Surgery, Fukushima Medical University, Fukushima, Japan; 13 Information Management and Statistics Office, Fukushima Medical University, Fukushima, Japan; 14 Department of Public Health, Dokkyo Medical University School of Medicine, Tochigi, Japan; 15 Department of Epidemiology, Radiation Effects Research Foundation, Hiroshima, Japan

## Abstract

Although the incidence of hepatobiliary enzyme abnormality increased immediately after the Great East Japan Earthquake and subsequent Fukushima Daiichi Nuclear Power Plant accident, longer-term trends remain unclear. The aims of this study were to determine longer-term trends in hepatobiliary enzyme abnormality and to elucidate lifestyle factors associated with such changes among residents of a nuclear-disaster-affected area. This longitudinal survey enrolled 20,395 adults living in the vicinity of Fukushima Daiichi Nuclear Power Plant. Data were obtained from the records of annual health checkups of adults aged ≥40 years between 2011 and 2012. Follow-up examinations were conducted from June 2013 to March 2014. Associations were assessed between changes in hepatobiliary enzyme abnormality immediately and 3–4 years after the disaster and lifestyle factors. The overall prevalence of hepatobiliary enzyme abnormality significantly decreased over the study period, from 29.9% to 27.1%. Multivariate logistic regression analysis revealed significant associations between improved hepatobiliary enzyme abnormality and improvements in daily physical activity and frequency of breakfast consumption. The results suggest that improvements in daily physical activity and frequency of breakfast consumption significantly reduced the incidence of hepatobiliary enzyme abnormality 3–4 years after the Great East Japan Earthquake and Fukushima Daiichi Nuclear Power Plant accident.

## Introduction

The Great East Japan Earthquake that occurred on March 11, 2011 off the northern Pacific coast of Japan was one of the largest ever recorded, with a magnitude of 9.0. The earthquake and subsequent tsunami caused a serious accident at the Fukushima Daiichi Nuclear Power Plant, forcing more than 160,000 residents of Fukushima Prefecture to evacuate. Immediately after the disaster, the Fukushima Health Management Survey (FHMS) was started to monitor the long-term health of residents^1^.

The early results of the FHMS have already shown increases in body weight and higher incidences of diabetes, dyslipidemia, atrial fibrillation, hypertension, renal dysfunction and metabolic syndrome among residents of the disaster-affected area^2–8^. According to the FHMS, a higher incidence of hepatobiliary enzyme abnormality was also observed immediately after the Great East Japan Earthquake, from 2011–2012^9^. We previously reported finding an association between increases in the incidence of hepatobiliary enzyme abnormality and evacuation due to the Fukushima Daiichi Nuclear Power Plant disaster^10^.

Although more than 5 years have passed since the disaster, more than 90,000 residents of Fukushima Prefecture have yet to return to their homes. Previous assessments of hepatobiliary enzyme abnormality using FHMS data were based on comparisons of incidences before and after the disaster^2–10^, and thus the results reflected relatively short-term effects; however, longer-term effects of the disaster on and lifestyle factors associated with hepatobiliary enzyme abnormality unclear.

Therefore, the objectives of the present study were to determine longer-term trends in hepatobiliary enzyme abnormality and to elucidate lifestyle factors associated with such changes among residents of Fukushima Prefecture 3–4 years after the Great East Japan Earthquake.

## Results

The overall incidence of hepatobiliary enzyme abnormality significantly decreased, from 29.9% in 2011–2012 to 27.1% in 2013–2014 (*p* < 0.001), and among all three groups (non-drinkers, 21.9% to 19.3%; light drinkers, 25.1% to 22.6%; and moderate/heavy drinkers, 49.8% to 46.1%) (Table 1). In addition, the incidence of moderate hepatobiliary enzyme abnormality in overall and all three groups also significantly decreased (overall, 6.9 to 5.9; non-drinkers, 3.8% to 3.1%; light drinkers, 4.7% to 3.8%; and moderate/heavy drinkers, 15.2% to 13.5%) (Supplement Table 1). In all three groups, the incidence of hypertension, dyslipidemia, and diabetes increased, while BMI significantly decreased. Moreover, the prevalence of obesity decreased overall and among non-drinkers.

**Table 1:** Clinical and biochemical characteristics of 20,395 participants classified by alcohol intake status in 2011-2012 and 2013-2014.

	Non-drinkers			Light drinkers			Moderate/Heavy drinkers			All		
	2011–2012	2013–2014	*p-value*	2011–2012	2013–2014	*p-value*	2011–2012	2013–2014	*p-value*	2011–2012	2013–2014	*p-value*
Number	6,264			9,315			4,816			20,395		
Sex (male/female)	1,231/5,033			3,543/5,772			4,245/571			9,019/11,376		
Age (years)	64.6 (7.7)	67.1 (7.8)		63.4 (8.0)	65.9 (8.0)		62.9 (7.8)	65.4 (7.9)		63.6 (7.9)	66.2 (7.9)	
Body weight (kg)	55.8 (10.1)	55.4 (10.3)	<0.001	58.1 (10.2)	57.8 (10.4)	<0.001	64.2 (10.1)	63.9 (10.3)	<0.001	58.8 (10.6)	58.5 (10.8)	<0.001
Body mass index (kg/m^2^)	23.7 (3.6)	23.6 (3.7)	0.007	23.69 (3.4)	23.65 (3.5)	<0.001	24.1 (3.1)	24.0 (3.2)	<0.001	23.8 (3.4)	23.7 (3.5)	<0.001
Overweight (≥25, %)	32.8	32.0	0.045	31.3	31.2	0.627	35.8	35.1	0.125	32.8	32.3	0.025
Smoking (yes)	7.2	6.7	0.001	10.2	9.5	<0.001	27.9	25.5	<0.001	13.5	12.4	<0.001
Hypertension (%)	53.1	54.5	0.005	50.1	51.3	0.001	62.0	63.2	0.032	53.9	55.1	<0.001
Dyslipidemia (%)	57.9	60.3	<0.001	51.5	56.0	<0.001	35.9	38.8	<0.001	49.8	53.3	<0.001
Diabetes (%)	10.9	13.8	<0.001	9.2	12.2	<0.001	13.0	16.4	<0.001	10.6	13.7	<0.001
AST (U/L)*	22 (19–26)	22 (19–26)	<0.001	23 (20–27)	23 (19–27)	<0.001	25 (21–31)	25 (21–30)	<0.001	23 (20–28)	23 (20–27)	<0.001
ALT (U/L)*	18 (14–24)	17 (14–23)	<0.001	18 (14–25)	18 (14–24)	<0.001	21 (16–30)	21 (16–28)	<0.001	19 (14–26)	18 (14–25)	<0.001
γ-GTP (U/L)*	19 (15–28)	19 (14–27)	<0.001	22 (16–34)	21 (16–31)	<0.001	41 (26–69)	38 (24–64)	<0.001	24 (16–39)	22 (16–36)	<0.001
hepatobiliary enzyme abnormality (%)	21.9	19.3	<0.001	25.1	22.6	<0.001	49.8	46.1	<0.001	29.9	27.1	<0.001

Data are mean values (standard deviation) or *median (interquartile range) for continuous variables, percentage values for categorical variables.

AST, aspartate aminotransferase; ALT, alanineaminotransferase; γ-GTP, gamma-glutamyl transpeptidase.

**Figure 1:**
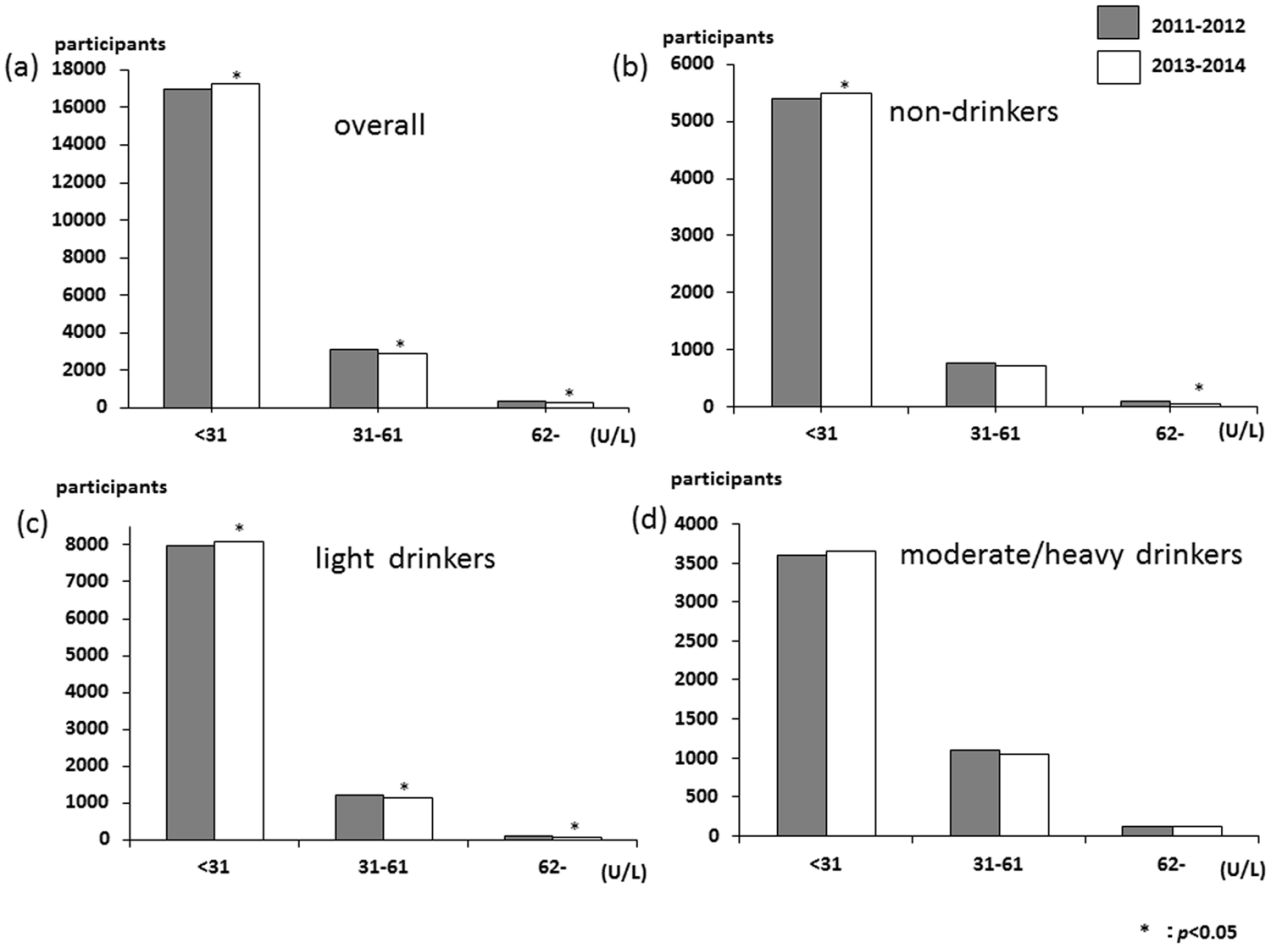
Distribution of serum AST (**a**) over all (n = 20395), (**b**) non-drinkers (n = 6264), (**c**) light drinkers (n = 9315), and (**d**) moderate/severe drinkers (n = 4816).

**Figure 2:**
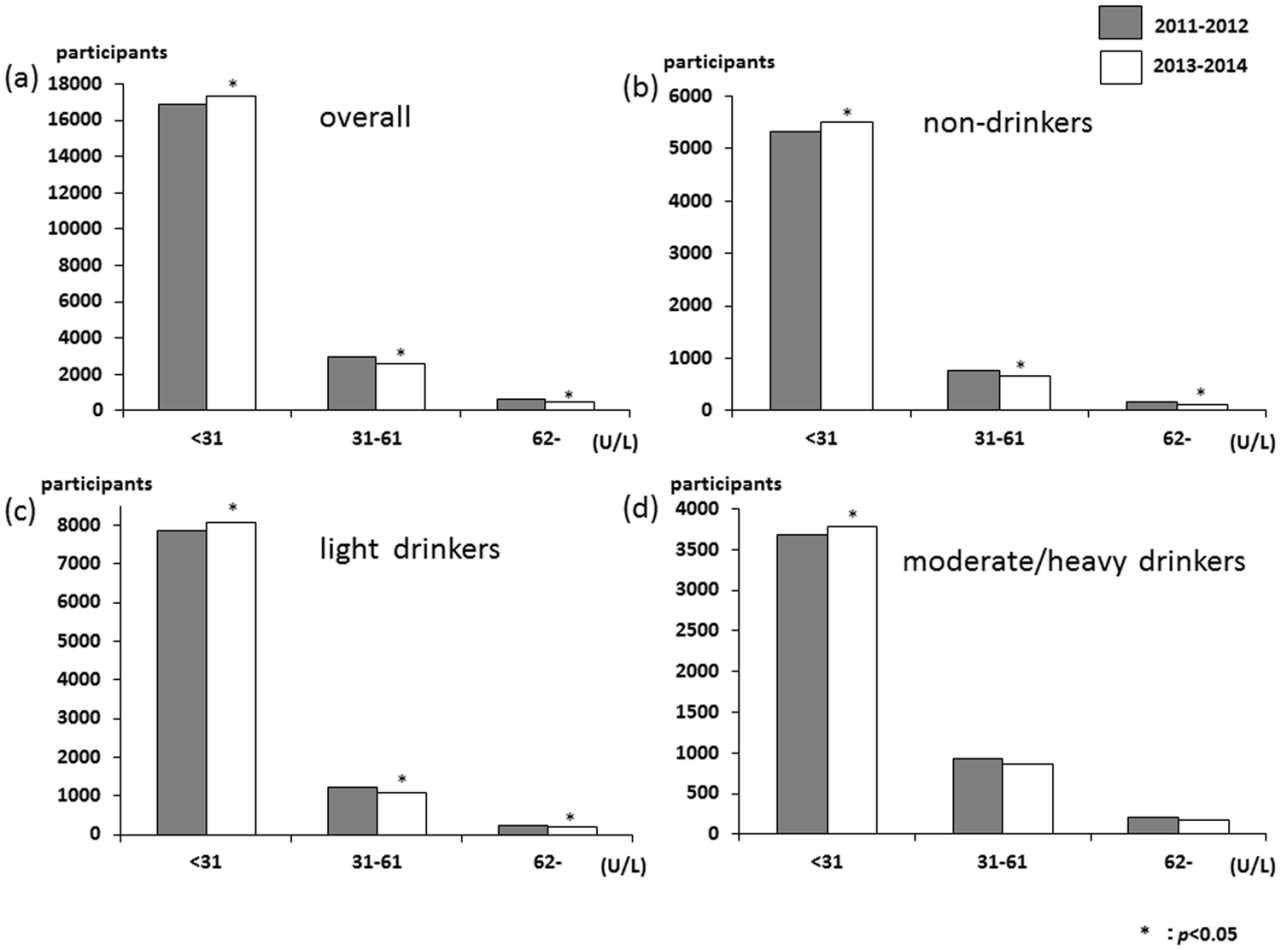
Distribution of serum ALT (**a**) over all (n = 20395), (**b**) non-drinkers (n = 6264), (**c**) light drinkers (n = 9315), and (**d**) moderate/severe drinkers (n = 4816).

**Figure 3:**
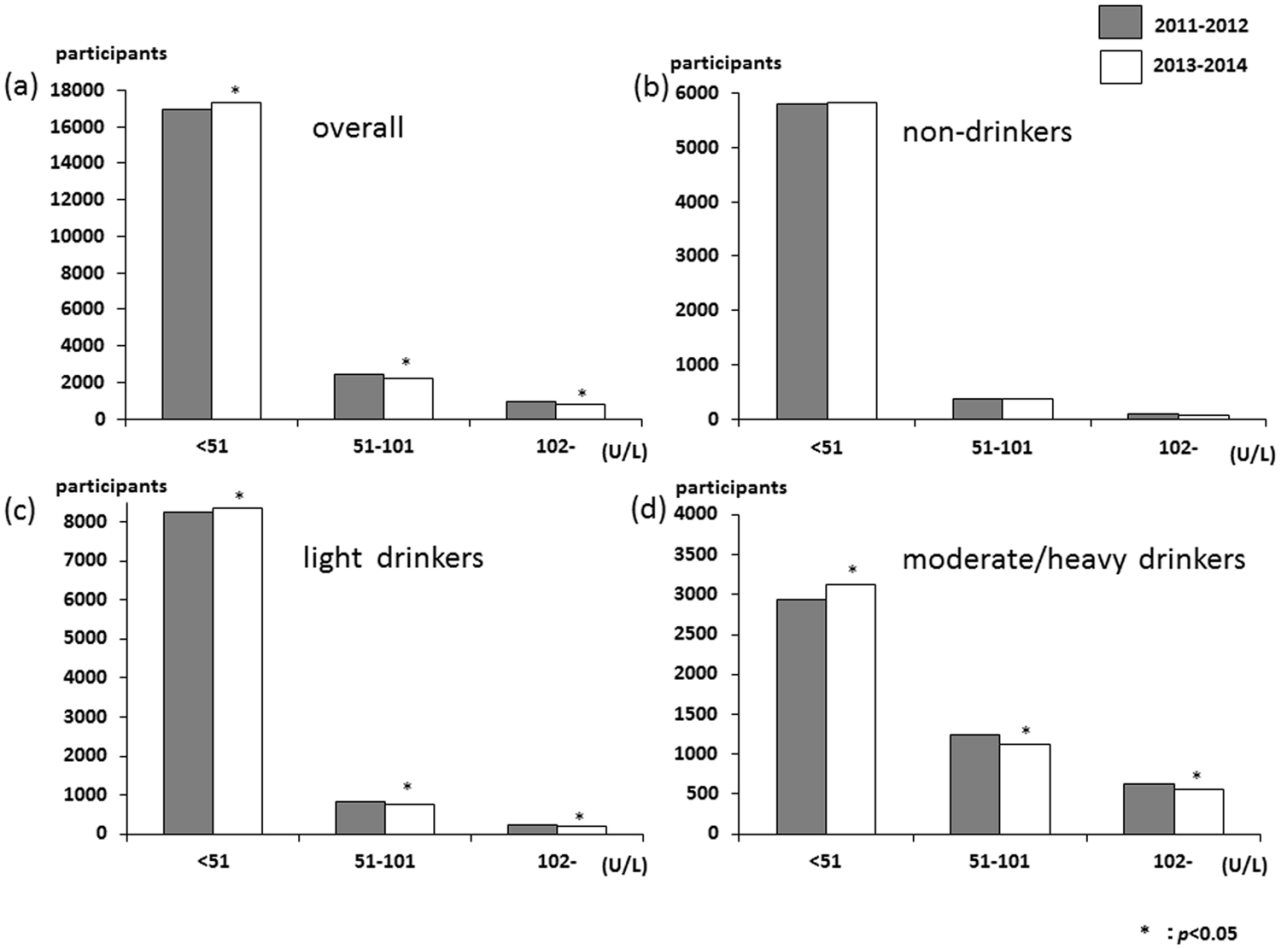
Distribution of serum γ-GTP (**a**) over all (n = 20395), (**b**) non-drinkers (n = 6264), (**c**) light drinkers (n = 9315), and (**d**) moderate/severe drinkers (n = 4816).

The distribution of the serum aspartate aminotransferase (AST) levels is shown in Fig. 1. AST levels were mostly mild, with about 98% of patients having AST levels less than 62 U/L. Only small numbers of participants were having AST levels higher than 61 U/L and the distribution increased in moderate/heavy drinkers (Fig. 1d). The overall proportion of AST levels more than 61 U/L significantly decreased, from 1.6% in 2011–2012 to 1.2% in 2013–2014 (*p* = 0.005), and among non- and light drinker groups (non-drinkers, 1.4% to 1.0%; and light drinkers, 1.2% to 0.8%). The distributions of serum alanine aminotransferase (ALT) and gamma-glutamyl transpeptidase (γ-GTP) levels are shown in Figs 2 and 3, respectively. These distributions were similar to that of AST levels. The overall proportion of ALT levels higher than 61 U/L significantly decreased, from 3.0% in 2011–2012 to 2.3% in 2013–2014 (*p* < 0.001), and among non- and light drinker groups (non-drinkers, 2.6% to 1.9%; and light drinkers, 2.6% to 2.0%). The overall proportion of γ-GTP levels more than 101 U/L significantly decreased, from 4.8% in 2011–2012 to 4.1% in 2013–2014 (*p* = 0.002), and among light and moderate/severe drinker groups (light drinkers, 2.7% to 2.2%; and moderate/heavy drinkers, 13.1% to 11.6%). The AST to ALT ratio in overall significantly increased, from 1.22 in 2011–2012 to 1.23 in 2013–2014 (*p* < 0.001), and among all three groups (non-drinkers, 1.25 to 1.27; light drinkers, 1.22 to 1.23; and moderate/heavy drinkers, 1.18% to 1.19). Moreover, the proportion of the AST to ALT ratio over 0.87 significantly increased in overall and all three groups (Supplement Table 1).

After the disaster, 1,648 (11.5%) of 14,288 participants without hepatobiliary enzyme abnormality in 2011–2012 were defined as having hepatobiliary enzyme abnormality in 2013–2014. The crude incidence rates (per 1000 person-years) of hepatobiliary enzyme abnormality in non-, light, and moderate/heavy drinkers were 35.7, 42.5, and 74.4, respectively. Risk factors of hepatobiliary enzyme abnormality in all three groups were male sex, increased BMI, and evacuation (Table 2). Other, risk factors of moderate hepatobiliary enzyme abnormality in all three groups were increased age, increased BMI, and evacuation (Supplement Table 2).

**Table 2:** Incidence rates and hazard ratios (95% confidence interval) of hepatobiliary enzyme abnormality for variables among 14,288 participants without hepatobiliary enzyme abnormality in 2011–2012.

	Non-drinkers		Light drinkers		Moderate/Heavy drinkers		All	
n of cases/N	442/4,893		752/6,976		454/2,419		1,648/14,288	
Crude incidence rate	35.7		42.5		74.4		45.5	
	HR (95% CI)	*p-value*	HR (95% CI)	*p-value*	HR (95% CI)	*p-value*	HR (95% CI)	*p-value*
Age (years)	0.99 (0.98–1.01)	0.332	1.00 (0.99–1.01)	0.839	0.99 (0.97–1.00)	0.025	0.99 (0.99–1.00)	0.053
Sex (male)	1.71 (1.36–2.15)	<0.001	1.63 (1.40–1.89)	<0.001	1.78 (1.30–2.44)	<0.001	1.82 (1.64–2.02)	<0.001
Body mass index (kg/m^2^)	1.07 (1.04–1.09)	<0.001	1.08 (1.06–1.10)	<0.001	1.08 (1.05–1.12)	<0.001	1.08 (1.06–1.09)	<0.001
Smoking (yes)	0.83 (0.56–1.22)	0.340	1.06 (0.83–1.36)	0.625	1.15 (0.92–1.42)	0.221	1.09 (0.94–1.26)	0.271
Evacuation (yes)	1.89 (1.56–2.28)	<0.001	2.14 (1.85–2.49)	<0.001	1.72 (1.42–2.09)	<0.001	1.95 (1.76–2.16)	<0.001
Alcohol intake (yes)	—	—	—	—	—	—	1.19 (1.07–1.34)	0.002

n indicates number; N, number of participants; CI, confidence interval.

Crude incidence rate (per 1000 person-years).

Multivariate logistic regression was used to investigate lifestyle factors associated with improvements in hepatobiliary enzyme abnormality among 18,070 participants (Table 3). After adjusting for age, sex, BMI, smoking status, evacuation and alcohol intake at baseline, significant associations were found between increases in daily physical activity and improved hepatobiliary enzyme abnormality. In addition, overall, more frequent breakfast consumption was significantly associated with improved hepatobiliary enzyme abnormality. Increased daily physical activity had also significant association with moderate hepatobiliary enzyme abnormality in non-drinkers (Supplement Table 3).

**Table 3:** Associations between improved hepatobiliary enzyme abnormality and changes in lifestyle factors among 18,070 participants through 2011–2012 to 2013–2014.

	Non-drinkers		Light drinkers		Moderate/Heavy drinkers		All	
	Odds ratio (95% CI)	*p-value*	Odds ratio (95% CI)	*p-value*	Odds ratio (95% CI)	*p-value*	Odds ratio (95% CI)	*p-value*
Daily physical activity (improved)	1.14 (1.11–1.78)	0.004	1.23 (1.02–1.50)	0.033	1.28 (1.00–1.63)	0.046	1.30 (1.15–1.48)	<0.001
Sleeping (improved)	0.99 (0.75–1.29)	0.924	1.00 (0.80–1.24)	0.967	0.99 (0.76–1.30)	0.970	0.99 (0.86–1.15)	0.934
Diet before bed time (improved)	0.96 (0.69–1.34)	0.812	0.94 (0.74–1.20)	0.615	1.13 (0.88–1.46)	0.347	1.00 (0.86–1.17)	0.961
Snack after dinner (improved)	0.83 (0.56–1.23)	0.348	1.04 (0.77–1.39)	0.814	0.83 (0.53–1.31)	0.428	0.92 (0.75–1.14)	0.456
Breakfast skipping (improved)	1.37 (0.80–2.32)	0.249	1.53 (1.04–2.25)	0.032	1.38 (0.85–2.25)	0.187	1.43 (1.10–1.86)	0.008
Eating speed (improved)	0.94 (0.66–1.32)	0.709	0.90 (0.68–1.18)	0.445	1.17 (0.87–1.58)	0.311	0.99 (0.83–1.17)	0.870

CI, confidence interval.

Adjusted for age, sex, body mass index, smoking, evacuation, and alcohol intake.

## Discussion

This study investigated trends associated with hepatobiliary enzyme abnormality after the Great East Japan Earthquake and subsequent Fukushima Daiichi Nuclear Power Plant accident. The results showed a decreased incidence of hepatobiliary enzyme abnormality 3–4 years after compared with immediately following the disaster for the first time. Male sex, increased BMI, and evacuation were identified as risk factors for the development of hepatobiliary enzyme abnormality. Conversely, increased levels of daily physical activity and frequency of breakfast consumption were lifestyle factors shown to be significantly associated with improved hepatobiliary enzyme abnormality. Notably, increased levels of daily physical activity was also significantly associated with improved moderate hepatobiliary enzyme abnormality in non-drinkers.

Previous cohort studies have reported finding elevated liver enzymes and an increased incidence of hepatobiliary enzyme abnormality after disasters^9–11^. Based on a longitudinal analysis, we recently reported that hepatobiliary enzyme abnormality was associated with evacuation after a disaster^10^. That study focused on the period before and immediately after a disaster, and therefore reflected the direct effects of a disaster on hepatobiliary enzyme abnormality. However, many evacuees have yet to return to their homes, and this likely affects their lifestyles. In the present study, we found that evacuation and increased BMI were risk factors for hepatobiliary enzyme abnormality. In addition, the decreased incidence of hepatobiliary enzyme abnormality among residents was a newly identified trend.

Metabolic syndrome and BMI are closely linked to hepatobiliary enzyme abnormality^12–15^. The incidence of both overweight and hepatobiliary enzyme abnormality decreased among non-drinkers. This finding suggests an association between increased BMI and hepatobiliary enzyme abnormality among non-drinkers. Obesity has been shown not only to induce fatty liver, but also to increase the prevalence of alcoholic liver disease^16^. Therefore, decreases in BMI may reduce the incidence of hepatobiliary enzyme abnormality, regardless of drinking status. In addition to hypertension and dyslipidemia, obesity is one of leading causes of metabolic syndrome. Interestingly, in this study, we found that the incidences of hypertension, dyslipidemia, and diabetes increased despite decreases in overweight. Although the precise reasons for this finding remain unclear, the incidence of these diseases may have been overestimated as a result of drug continuation. Another possible reason could have been differences in disease mechanisms or the effect of overweight on hepatobiliary enzyme abnormality and those associated diseases.

Lifestyles were dramatically altered after the Great East Japan Earthquake and subsequent Fukushima Daiichi Nuclear Power Plant accident. To our knowledge, this is the first study to investigate the effects of various lifestyle factors on hepatobiliary enzyme abnormality. Physical activity or exercise has been reported to be associated with hepatobiliary enzyme abnormality^17–19^. The results of the present study support an association between increases in physical activity and improvements in hepatobiliary enzyme abnormality even in unusual life after the Great East Japan Earthquake and subsequent Fukushima Daiichi Nuclear Power Plant accident. Moreover, present study showed an association between increases in physical activity and improvements in moderate hepatobiliary enzyme abnormality in non-drinkers. In fact, the proportion of residents who reported walking or exercising more than 1 hour per day increased form 33.4% in 2011–2012 to 35.0% in 2013–2014. This finding may reflect effective efforts to promote exercise by municipal governments. Although no association was found between improved sleep and hepatobiliary enzyme abnormality in the present study, short sleep duration has been reported to be associated with metabolic syndrome and health outcomes^20, 21^. Therefore, a more detailed analysis of sleep disorder may reveal associations with hepatobiliary enzyme abnormality after a disaster.

Changes in the living environment can increase the prevalence of mental health issues. The FHMS reported finding severe traumatic events among evacuees^22^. Mental disorders may increase the prevalence of hepatobiliary enzyme abnormality because obesity is associated with psychiatric disorders^23^. The incidence of psychological distress and post-traumatic stress decreased over time among residents in the evacuation zone after the Fukushima Daiichi Nuclear Power Plant accident^24^. Therefore, improved mental health after a disaster may promote a decrease in the incidence of hepatobiliary enzyme abnormality.

The present study did have several limitations. First, it was based only on an analysis of routine health checkups. Therefore, liver function such as Child-Pugh score or liver fibrosis scoring such as AST to platelet ratio index^25^ and FIB-4 index^26^ could not be evaluated. Other, we confirmed significant increase in the AST to ALT ratio, however, this ratio can be affected by age^27^. Moreover, no definitive diagnoses of hepatobiliary enzyme abnormality were established. In future studies, it will be critical to identify the precise cause of hepatobiliary enzyme abnormality, for example, viral hepatitis or fatty liver and medication. Second, the evaluation of lifestyle factors in the present study was based on responses to questionnaire surveys during health checkups, and therefore not very comprehensive. The associated dynamics in use of healthcare resources, such as antidiabetics, other related drugs and alcohol rehabilitation could not be evaluated in present study. Moreover, we could not evaluate food intake, mental status, or sleep duration and quality, all of which can affect obesity and hepatobiliary enzyme abnormality. It will be important in the future to elucidate which lifestyle factors are most affected by evacuation, as this could help in the development of novel strategies for protecting against the onset of hepatobiliary enzyme abnormality after a disaster. Third, there were no comparative cohorts in the present study. Future research is needed to compare the changes in hepatobiliary enzyme during the same observation period between residents living in the evacuation area and those living in an area far from the Fukushima Daiichi Nuclear Power plant.

In conclusion, in parallel with decreases in BMI, the incidence of hepatobiliary enzyme abnormality decreased 3–4 years after compared with immediately after the Great East Japan Earthquake. Improvements in lifestyle factors such as daily physical activity and breakfast consumption were associated with improved hepatobiliary enzyme abnormality. These findings are expected to be important for periodic health checkups and lifestyle recommendations among individuals at risk of developing hepatobiliary enzyme abnormality, regardless of evacuation status.

## Methods

### Study population

**Figure 4:**
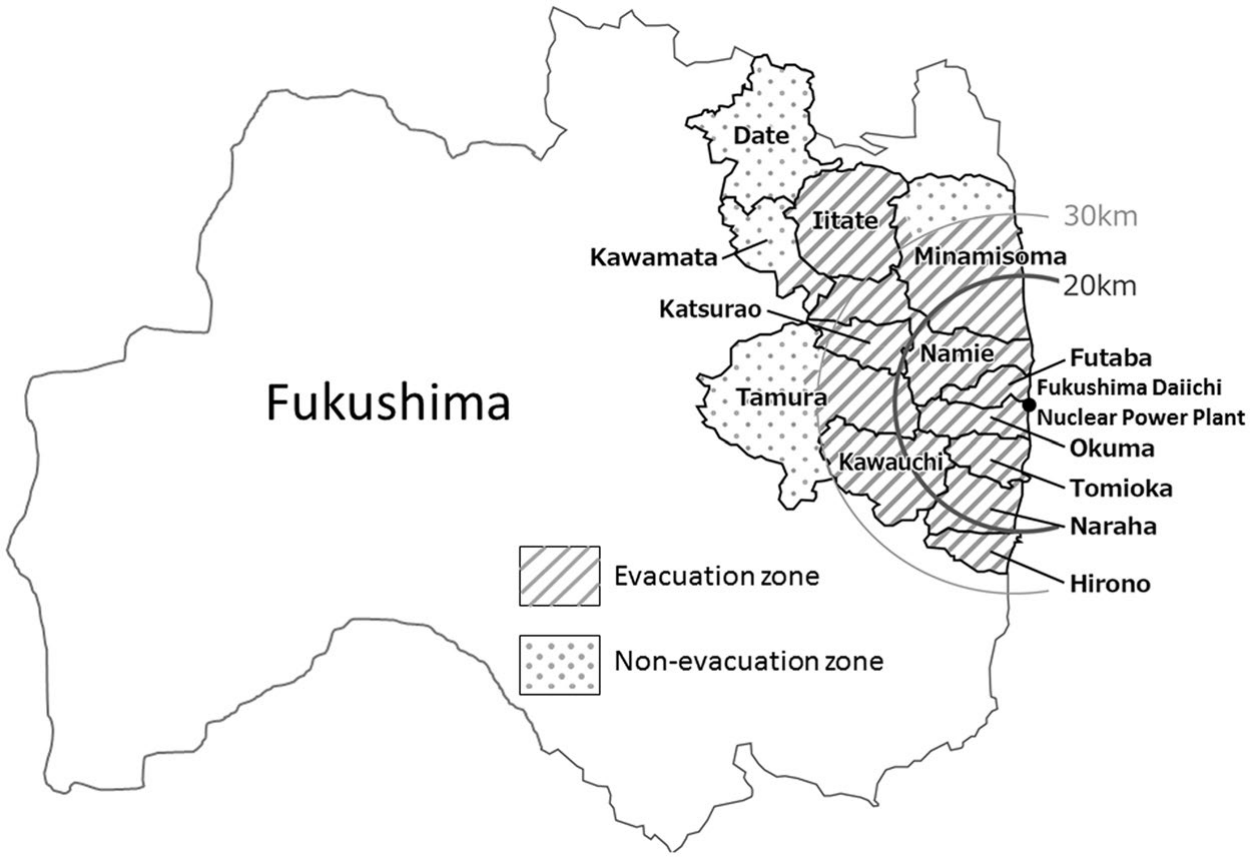
Map showing the location of the Fukushima Daiichi Nuclear Power Plant, the evacuation zone, and the non-evacuation zone. The map was created by authors using Adobe Illustrator CS6 and Microsoft Power point 2010 software based on the map of Assistance of Residents Affected by the Nuclear Incidents (Ministry of Economy, Trade and Industry of Japan) (http://www.meti.go.jp/english/earthquake/nuclear/roadmap/pdf/150905Map).

For the purposes of this study, we analyzed a subset of participants from the FHMS. The participants were Japanese adults living in the following municipalities of Fukushima Prefecture at the time of the Fukushima Daiichi Nuclear Power Plant: Tamura city; Minamisoma city; Kawamata town; Hirono town; Naraha town; Tomioka town; Kawauchi village; Okuma town; Futaba town; Namie town; Katsurao village; Iitate village; and Date city. All residents of Hirono town, Naraha town, Tomioka town, Kawauchi village, Okuma town, Futaba town, Namie town, Katsurao village, Iitate village, and parts of Tamura city, Minamisoma city, Kawamata town, and Date city were forced to evacuate by government order based on increased local radiation levels after the Fukushima Daiichi Nuclear Power Plant accident (Fig. 4). In these communities, adult residents aged 40–74 years and elderly residents aged ≥75 years (the target population for health checkups comprised 18,745 men and 22,888 women; mean age, 67 years) underwent comprehensive annual health checkups between June 2011 and March 2013. Follow-up health checkups were conducted from June 2013 to March 2014. Detailed methods of the comprehensive health checkups have been published previously^1^. Follow-up surveys for the FHMS were conducted nationwide because the evacuees had relocated to various parts of the country. In the evaluation of lifestyle factors, this study limited all analyses to adults aged <75 years at the time of follow-up health checkups. A total of 20,974 adults (9,369 men, 11,605 women) underwent follow-up examinations after the disaster (mean time of follow up: 2.5 years). If a participant underwent a health checkup more than once during each survey period (immediately after [2011–2012] and 3–4 years later [2013–2014]), we used the data from the earliest examination in 2011–2012 and the latest examination in 2013–2014 to most accurately assess the long-term effects of the disaster on health status. A total of 579 participants with liver disease and insufficient data for assessing liver function were excluded. Finally, 20,395 participants (9,019 men, 11,376 women) were eligible for analysis in this study.

Informed consent was obtained from all subjects and community representatives to conduct an epidemiologic study based on the guidelines of the Council for International Organizations of Medical Science^28^. This study protocol was reviewed and approved by the Ethics Committee of Fukushima Medical University (#1916). The study was conducted in accordance with the approved guidelines.

### Biochemical analysis

The following laboratory data were obtained from all participants: AST, U/L; ALT, U/L; γ-GTP, U/L; high-density lipoprotein cholesterol (HDL-C, mg/dL); low-density lipoprotein cholesterol (LDL-C, mg/dL); triglycerides (TG, mg/dL); fasting plasma glucose (FPG, mg/dL); and HbA1c (%). HbA1c values were estimated based on National Glycohemoglobin Standardization Program units, and the equivalent value was calculated using the following equation: HbA1c (%) = 1.019 × HbA1c (Japanese Diabetes Society) (%) +0.4%^29^. Hepatobiliary enzyme abnormality was defined as AST ≥ 31 U/L, ALT ≥ 31 U/L, or γ-GTP ≥ 51 U/L. In addition, moderate hepatobiliary enzyme abnormality was defined as AST ≥ 62 U/L, ALT ≥ 62 U/L, or γ-GTP ≥ 102 U/L. Liver fibrosis was evaluated using AST to ALT ratio, moreover, 0.87 was used as a cut off based on the Japanese criteria^30^. Diabetes was defined as fasting plasma glucose ≥ 126 mg/dL, HbA1c ≥ 6.5%, or the self-reported use of antihyperglycemic agents. Dyslipidemia was defined as fasting LDL-C ≥ 140 mg/dL, fasting TG ≥ 150 mg/dL, fasting HDL-C < 40 mg/dL, or the self-reported use of antidyslipidemic agents.

### Assessment of other variables

Height in stocking feet and weight in light clothing were measured, and body mass index (BMI) was calculated as weight in kilograms divided by height in meters squared. Overweight was defined as a BMI ≥ 25 kg/m^2^. Systolic and diastolic blood pressure (BP) values were measured by trained technicians using a standard mercury sphygmomanometer on the right arm of seated participants. Hypertension was defined as systolic BP > 140 mmHg, diastolic BP > 90 mmHg, or the self-reported use of antihypertensive agents. Interviews were conducted to obtain the history of cigarette smoking, weekly alcohol intake, and medication history. Participants were classified into the following three groups according to alcohol consumption: (1) non-drinkers, defined as drinking no alcohol; (2) light drinkers, defined as drinking ≤22 g of alcohol per day; or (3) moderate/heavy drinkers, defined as drinking >22 g of alcohol per day.

### Statistical analysis

Changes in data immediately and 3–4 years after the disaster were compared using the paired Student’s t test and Wilcoxon signed-rank test. For participants with no liver dysfunction at baseline (immediately after the disaster), we tested associations between evacuation and other potential confounders with the incidence of liver dysfunction after the disaster using the Cox proportional hazards model. The following variables were considered potential confounding factors: age (continuous), sex, BMI (continuous), current smoking (yes or no), evacuation (yes or no), alcoholic intake (yes or no).

In addition, odds ratios and 95% confidence intervals were calculated to analyze the association between improvements in liver dysfunction and changes in lifestyle factors using a logistic regression model. Because alcohol consumption is one of the major causes of liver dysfunction, analysis was stratified according to drinking status (non-drinkers, light drinkers, or moderate/heavy drinkers). SAS version 9.3 (SAS Institute, Cary, NC, USA) was used for all statistical analyses. All probability values for statistical tests were two-tailed, and *p* values < 0.05 were considered statistically significant.

## Electronic supplementary material

supplement table
