# Supplementary material for: Changes in Hepatobiliary Enzyme Abnormality After the Great East Japan Earthquake: The Fukushima Health Management Survey
Source: Sci Rep. 2017 Apr 6;7:710. doi: 10.1038/s41598-017-00776-7 (PMC5429598; doi:10.1038/s41598-017-00776-7)
Supplement: Supplementary file 1 — supplement table [file 41598_2017_776_MOESM1_ESM.pdf]

Changes in Hepatobiliary Enzyme Abnormality After the Great East Japan Earthquake: The Fukushima Health Management Survey

Atsushi Takahashi,[1,2,3] Tetsuya Ohira,[1,2,4] Mayu Uemura,[2,4] Mitsuaki Hosoya,[1,2,5] Seiji Yasumura,[1,2,6] Shigeatsu Hashimoto,[1,2,7] Hiromasa Ohira,[2,3] Akira Sakai,[1,2,8] Akira Ohtsuru,[1,2,9] Hiroaki Satoh,[1,2,7] Yukihiko Kawasaki,[1,2,5] Hitoshi Suzuki,[1,2,10] Yoshihiro Sugiura,[1,2,11] Hiroaki ShiShido,[1,2,12] Yoshimitsu Hayashi,[1,2,7] Hideto Takahashi,[1,13] Hironori Nakano,[1,2,4] Gen Kobashi,[1,14] Kotaro Ozasa,[1,15] Hitoshi Ohto,[1,2] and Masafumi Abe[1,2]

[1]Radiation Medical Science Center for the Fukushima Health Management Survey; Office of the Comprehensive Health Check and Health Promotion, [2]Fukushima Medical University School of Medicine, Fukushima, Japan; Department of [3]Gastroenterology, [4]Epidemiology, [5]Pediatrics, [6]Public Health, [7] Nephrology, Hypertension, Diabetology, and Endocrinology, [8]Radiation Life Sciences, [9]Radiation Health Management, [10]Cardiology, [11]Neurology, [12]Orthopaedic Surgery, [13]Information Management and Statistics Office, [14]Department of Public Health, Dokkyo Medical University School of Medicine, Tochigi, Japan, [15]Department of Epidemiology, Radiation Effects Research Foundation, Hiroshima, Japan

Running title: Changes in Hepatobiliary Enzyme Abnormality After the Great East

Japan Earthquake

Correspondence to: Atsushi Takahashi, M.D., Department of Gastroenterology, Fukushima Medical University, 1 Hikarigaoka, Fukushima 960-1295, Japan

E-mail: junior@fmu.ac.jp, Telephone: +81-24-547-1202; Fax: +81-24-547-2005

Supplement Table 1. Clinical and biochemical characteristics of 20,395 participants classified by alcohol intake status in 2011-2012 and 2013-2014.

	Non-drinkers			Light drinkers			Moderate/Heavy drinkers			All		
	2011-2012	2013-2014	*p-value*	2011-2012	2013-2014	*p-value*	2011-2012	2013-2014	*p-value*	2011-2012	2013-2014	*p-value*
Number	6,264			9,315			4,816			20,395		
Sex (male/female)	1,231 / 5,033			3,543 / 5,772			4,245 / 571			9,019 / 11,376		
Age (years)	64.6 (7.7)	67.1 (7.8)		63.4 (8.0)	65.9 (8.0)		62.9 (7.8)	65.4 (7.9)		63.6 (7.9)	66.2 (7.9)	
Body weight (kg)	55.8 (10.1)	55.4 (10.3)	<0.001	58.1 (10.2)	57.8 (10.4)	<0.001	64.2 (10.1)	63.9 (10.3)	<0.001	58.8 (10.6)	58.5 (10.8)	<0.001
Body mass index (kg/m^2)	23.7 (3.6)	23.6 (3.7)	0.007	23.69 (3.4)	23.65 (3.5)	<0.001	24.1 (3.1)	24.0 (3.2)	<0.001	23.8 (3.4)	23.7 (3.5)	<0.001
Overweight (≥25, %)	32.8	32.0	0.045	31.3	31.2	0.627	35.8	35.1	0.125	32.8	32.3	0.025
Smoking (yes)	7.2	6.7	0.001	10.2	9.5	<0.001	27.9	25.5	<0.001	13.5	12.4	<0.001
Hypertension (%)	53.1	54.5	0.005	50.1	51.3	0.001	62.0	63.2	0.032	53.9	55.1	<0.001
Dyslipidemia (%)	57.9	60.3	<0.001	51.5	56.0	<0.001	35.9	38.8	<0.001	49.8	53.3	<0.001
Diabetes (%)	10.9	13.8	<0.001	9.2	12.2	<0.001	13.0	16.4	<0.001	10.6	13.7	<0.001
AST (U/L)*	22 (19-26)	22 (19-26)	<0.001	23 (20-27)	23 (19-27)	<0.001	25 (21-31)	25 (21-30)	<0.001	23 (20-28)	23 (20-27)	<0.001
ALT (U/L)*	18 (14-24)	17 (14-23)	<0.001	18 (14-25)	18 (14-24)	<0.001	21 (16-30)	21 (16-28)	<0.001	19 (14-26)	18 (14-25)	<0.001
γ-GTP (U/L)*	19 (15-28)	19 (14-27)	<0.001	22 (16-34)	21 (16-31)	<0.001	41 (26-69)	38 (24-64)	<0.001	24 (16-39)	22 (16-36)	<0.001
Moderate hepatobiliary enzyme abnormality (%)	3.8	3.1	0.011	4.7	3.8	<0.001	15.2	13.5	0.001	6.9	5.9	<0.001
AST/ALT ratio	1.25 (1.00-1.50)	1.27 (1.00-1.53)	<0.001	1.22 (1.00-1.47)	1.23 (1.00-1.47)	<0.001	1.18 (0.95-1.44)	1.19 (0.98-1.46)	<0.001	1.22 (1.00-1.47)	1.23 (1.00-1.50)	<0.001
AST/ALT ≥ 0.87 (%)	85.9	88.0	<0.001	84.5	86.6	<0.001	82.4	85.0	<0.001	84.4	86.6	<0.001

Data are mean values (standard deviation) or *median (interquartile range) for continuous variables, percentage values for categorical variables.

AST, aspartate aminotransferase; ALT, alanineaminotransferase; γ-GTP, gamma-glutamyl transpeptidase

Supplement Table 2. Incidence rates and hazard ratios (95% confidence interval) of moderate hepatobiliary enzyme abnormality for variables among 18,987 participants without hepatobiliary enzyme abnormality in 2011-2012.

	Non-drinkers		Light drinkers		Moderate/Heavy drinkers		All	
n of cases/ N	123 / 6,028		167 / 8,873		231 / 4,086		521 / 18,987	
Crude incidence rate	8.1		7.5		22.5		10.9	
	HR (95% CI)	*p-value*	HR (95% CI)	*p-value*	HR (95% CI)	*p-value*	HR (95% CI)	*p-value*
Age (years)	0.96 (0.94-0.98)	<0.001	0.97 (0.96-0.99)	0.003	0.97 (0.96-0.99)	0.003	0.97 (0.96-0.98)	<0.001
Sex (male)	1.36 (0.87-2.11)	0.177	1.41 (1.02-1.95)	0.038	1.76 (1.08-2.89)	0.024	2.01 (1.65-2.45)	<0.001
Body mass index (kg/m^2)	1.14 (1.10-1.19)	<0.001	1.10 (1.07-1.13)	<0.001	1.12 (1.07-1.17)	<0.001	1.10 (1.08-1.12)	<0.001
Smoking (yes)	1.08 (0.57-2.05)	0.818	1.33 (0.85-2.09)	0.215	1.37 (1.03-1.84)	0.032	1.38 (1.10-1.73)	0.006
Evacuation (yes)	2.72 (1.89-3.92)	<0.001	3.55 (2.58-4.90)	<0.001	2.51 (1.93-3.27)	<0.001	2.85 (2.39-3.41)	<0.001
Alcohol intake (yes)	-	-	-	-	-	-	1.11 (0.90-1.38)	0.329

n indicates number; N, number of participants; CI, confidence interval.

Crude incidence rate (per 1000 person-years).

Supplement Table 3. Associations between improved moderate hepatobiliary enzyme abnormality and changes in lifestyle factors among 18,070 participants through 2011-2012 to 2013-2014.

	Non-drinkers		Light drinkers		Moderate/Heavy drinkers		All	
	Odds ratio (95% CI)	*p-value*	Odds ratio (95% CI)	*p-value*	Odds ratio (95% CI)	*p-value*	Odds ratio (95% CI)	*p-value*
Daily physical activity (improved)	1.87 (1.26-2.78)	0.002	0.79 (0.52-1.18)	0.245	1.11 (0.79-1.56)	0.544	1.13 (0.91-1.40)	0.269
Sleeping (improved)	0.56 (0.31-1.00)	0.048	0.93 (0.61-1.40)	0.710	1.07 (0.75-1.53)	0.723	0.88 (0.69-1.13)	0.313
Diet before bed time (improved)	1.29 (0.76-2.19)	0.346	1.23 (0.82-1.86)	0.323	0.73 (0.49-1.08)	0.112	1.00 (0.78-1.29)	0.996
Snack after dinner (improved)	1.26 (0.69-2.31)	0.448	0.53 (0.27-1.05)	0.068	1.10 (0.62-1.95)	0.741	0.85 (0.60-1.21)	0.373
Breakfast skipping (improved)	0.40 (0.10-1.68)	0.210	1.14 (0.56-2.31)	0.717	1.16 (0.61-2.21)	0.648	0.95 (0.61-1.48)	0.817
Eating speed (improved)	0.99 (0.55-1.79)	0.981	1.20 (0.76-1.87)	0.431	1.24 (0.84-1.83)	0.283	1.17 (0.90-1.52)	0.245

CI, confidence interval.

Adjusted for age, sex, body mass index, smoking, evacuation, and alcohol intake.
